# A Comparison of Nutritional Antioxidant Content in Breast Milk, Donor Milk, and Infant Formulas

**DOI:** 10.3390/nu8110681

**Published:** 2016-10-28

**Authors:** Corrine Hanson, Elizabeth Lyden, Jeremy Furtado, Matthew Van Ormer, Ann Anderson-Berry

**Affiliations:** 1 College of Allied Health Professions, University of Nebraska Medical Center, Medical Nutrition Education, 984045 Nebraska Medical Center, Omaha, NE 68198-4045, USA; 2 College of Public Health, University of Nebraska Medical Center, 984375 Nebraska Medical Center, Omaha, NE 68198-4375, USA; elyden@unmc.edu; 3 Department of Nutrition, Harvard School of Public Health, 655 Huntington Avenue, Boston, MA 02215, USA; jfurtado@hsph.harvard.edu; 4 Pediatrics, University of Nebraska Medical Center, 981205 Nebraska Medical Center, Omaha, NE 68198-1205, USA; Matthew.vanormer@unmc.edu (M.V.O.); alanders@unmc.edu (A.A.-B.)

**Keywords:** antioxidants, breast milk, infant feeding, infant formula, breast milk substitutes, human milk

## Abstract

Human milk is the optimal food for human infants, including infants born prematurely. In the event that a mother of a hospitalized infant cannot provide breast milk, donor milk is considered an acceptable alternative. It is known that the macronutrient composition of donor milk is different than human milk, with variable fat content and protein content. However, much less is known about the micronutrient content of donor milk, including nutritional antioxidants. Samples of breast milk from 12 mothers of infants hospitalized in the Newborn Intensive Care Unit until were collected and analyzed for concentrations of nutritional antioxidants, including α-carotene, β-carotene, β-cryptoxanthin, lycopene, lutein + zeaxanthin, retinol, and α-tocopherol. Additionally, a homogenized sample of donor milk available from a commercial milk bank and samples of infant formulas were also analyzed. Concentrations of nutritional antioxidants were measured using high-performance liquid chromatography. Compared to breast milk collected from mothers of hospitalized infants, commercially available donor milk had 18%–53% of the nutritional antioxidant content of maternal breast milk. As donor milk is becoming a common nutritional intervention for the high risk preterm infant, the nutritional antioxidant status of donor milk–fed premature infants and outcomes related to oxidative stress may merit further investigation.

## 1. Introduction

Human milk is the optimal food for infants, including infants born prematurely. In the event that a mother of a hospitalized infant cannot provide breast milk, donor milk is often considered an acceptable or even ideal alternative to the mother’s own milk. It is known that the macronutrient composition of donor milk is different from human milk, with variable fat content and protein content lower than that of mature milk [1,2,3,4]. However, much less is known about the micronutrient content of donor milk, including nutritional antioxidants.

There is increasing evidence that links early exposure to oxidative stress with potentially lifelong consequences. The premature infant is especially susceptible to damage from oxidative stress for two reasons: (1) adequate concentrations of antioxidants may be absent at birth; (2) the ability to increase synthesis of antioxidants is impaired. This can lead to an increased risk for the development of oxidative stress–induced diseases such as bronchopulmonary dysplasia (BPD), retinopathy of prematurity (ROP), necrotizing enterocolitis (NEC), and periventricular leukomalacia (PVL) [5,6]. Therefore, it is critical in premature infants to ensure an adequate supply of dietary antioxidants. The objective of this analysis was to compare the nutritional antioxidant profile of different types of feedings for premature infants, including samples of maternal breast milk collected during neonatal hospitalization and pasteurized pooled donor milk.

## 2. Materials and Methods

A total of 12 breast milk samples from women with singleton infants who were admitted to the Neonatal Intensive Care Unit were collected for analysis. These samples were obtained as a subset of subjects (*n* = 30) who were enrolled in a study of nutritional antioxidant status of Newborn Intensive Care Unit (NICU) hospitalized infants who had excess breast milk available after clinical use. Institutional Review Board approval was obtained prior to collection of any samples. The median gestational age was 37.1 weeks, with a range 30.3–42.0 weeks. A 2.0 mL sample was collected from each participant in a sterile plastic tube, protected from heat and light, and stored at −80 °F freezers until they were analyzed. In addition, a single 2 mL aliquot of the commercially available, pooled donor milk sample was collected for analysis, and single 2 mL samples of the commercially available preterm infant formula, transitional infant formula, and term infant formula used in the study unit were collected. All formulas were from a single manufacturer (Abbott Nutrition^®^). Analysis of samples was performed at the Biomarker Research Institute at the Harvard School of Public Health. Measurements of lutein + zeaxanthin, β-cryptoxanthin, *trans*-lycopene, *cis*-lycopene, total lycopene, α-carotene, *trans*-β-carotene, *cis*-β-carotene, total-β-carotene, retinol, α-tocopherol and γ-tocopherol were obtained. Concentrations in plasma samples were measured as described by El-Sohemy et al [7]. Plasma samples (250 µL) were mixed with 250 mL ethanol containing 10 µg *rac*-tocopherol/mL (Tocol) as an internal standard, extracted with 4 mL hexane, evaporated to dryness under nitrogen, and reconstituted in 100 mL ethanol-dioxane (1:1, by vol) and 150 mL acetonitrile. Samples are quantitated by high-performance liquid chromatography (HPLC) on a Restek Ultra C_18_ 150 mm × 4.6 mm column with a 3 µm particle size encased in a column oven (Hitachi L-2350, Hitachi, San Jose, CA, USA) to prevent temperature fluctuations, and equipped with a trident guard cartridge system (Restek, Corp., Bellefonte, PA, USA). A mixture of acetonitrile, tetrahydrofuran, methanol, and a 1% ammonium acetate solution (68:22:7:3) was used as the mobile phase at a flow rate of 1.1 mL/min, with a Hitachi L-2130 pump in isocratic mode, a Hitachi L-2455 diode array detector (300 nm and 445 nm), and a Hitachi L-2200 auto-sampler with water-chilled tray. The Hitachi System Manager software (D-2000 Elite, Version 3.0) was used for peak integration and data acquisition. Because lutein and zeaxanthin co-elute on the chromatogram, the two are grouped and provided as lutein + zeaxanthin. Internal quality control was monitored with four control samples analyzed within each run. These samples consisted of two identical high-level plasmas and two identical low-level plasmas. Comparison of data from these samples allowed for within-run and between-run variation estimates. In addition, external quality control was monitored by participation in the standardization program for carotenoid analysis from the National Institute of Standards and Technology U.S.A. Descriptive statistics included means and standard deviations. A one sample *t*-test was used to compare the mean value from the 12 maternal breast milk (MBM) to the known value of the donor or infant formulas values *p* < 0.05 was considered statistically significant.

## 3. Results

The results for the concentrations of α-carotene, total β-carotenes, β-cryptoxanthin, total lycopenes, lutein + zeaxanthin, retinol, α-tocopherol, and γ-tocopherol for each of the feeding types are shown in Table 1.

**Figure 1:**
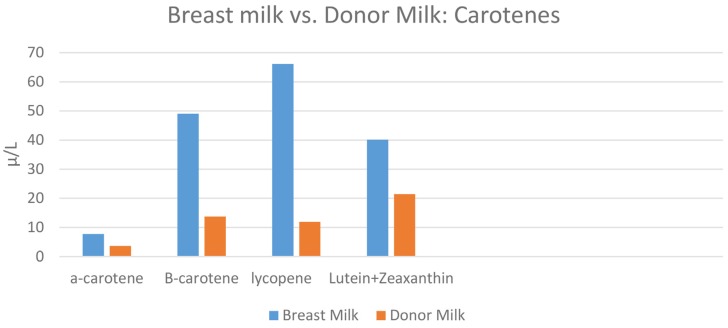
The concentrations of α-carotene, β-carotene, lycopene, and lutein + zeaxanthin in maternal breast milk vs. donor milk samples. Lycopene was statistically significant (*p* = 0.006).

When the concentrations of carotenoids between the 12 breast milk samples and the pooled donor milk sample were compared, the donor milk sample was descriptively lower in all carotenoids. A statistically significant difference was found between concentrations of total lycopene (*p* = 0.006). A comparison of levels of carotenoids breast milk vs. donor milk is shown in Figure 1.

Samples of the transitional formula and premature formula were also significantly lower in lycopene when compared to breast milk (*p* = 0.003 and 0.002, respectively) (see Table 1).

**Figure 2:**
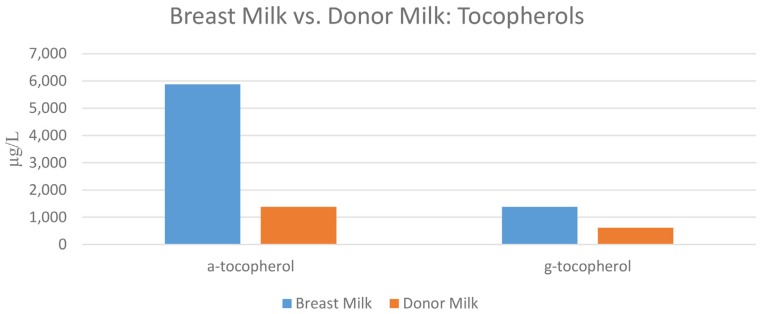
The concentrations of α-tocopherol and γ-tocopherol in maternal breast milk vs. donor milk samples. All values were significantly different (*p* = 0.009 and 0.01 for α-tocopherol and γ-tocopherol, respectively).

When concentrations of tocopherols between the 12 breast milk samples and the pooled donor milk sample were compared, a statistically significant difference was found between concentrations of both α- and γ-tocopherols (*p* = 0.009 and 0.01, respectively). A comparison of concentrations of tocopherols in breast milk vs. donor milk is shown in Figure 2.

Samples of the transitional formula and premature formula were significantly higher in α-tocopherol when compared to breast milk (*p* = 0.003 and 0.002) and all infant formulas were significantly higher in γ-tocopherol when compared to breast milk (*p* ˂ 0.0001 for term, transitional, and premature formulas) (see Table 1).

## 4. Discussion

Donor milk is considered to be an effective alternative source of nutrition when the mother’s own milk is not available, and preterm infants are the primary recipients. Donor milk is obtained from healthy, lactating mothers who consent to donate their surplus which is collected, processed, and stored by specialized centers such as human milk banks. Donor milk is pasteurized to reduce microbial growth and ensure its safety for consumption. The most common pasteurization procedure is Holder pasteurization, in which milk is exposed to a temperature of approximately 62.5 °C (144.5 °F) for at least 30 min [8]. Pasteurization is necessary to inactivate most viral and bacterial compounds, but can affect the nutrition and immunological properties of breast milk. While it has been shown that pasteurized mother’s milk retains some of the beneficial and protective effects [1,4,9] there does appear to be an impact on the antioxidant capacity of donor milk [10,11]. Significant decreases in the anti-oxidant compounds malondialdehye and glutathione have been found after pasteurization [12]. The pasteurization of human milk has also been shown to result in significant losses of vitamin D, with reductions of 10%–20% [13].

Preterm infants are born relatively deficient in antioxidant defenses, with increased oxidant stress [5]. Many events, such as infection, mechanical ventilation, intravenous nutrition, and blood transfusions result in oxidative stress. Oxidative stress is associated with serious conditions in the newborn, such as bronchopulmonary dysplasia (BPD), respiratory distress, retinopathy of prematurity (ROP), and necrotizing enterocolitis (NEC), as well as an increased risk of infection [6]. Ensuring adequate nutritional antioxidant status may provide protective benefits to infants at an increased risk of developing these conditions or may positively impact an infant’s recovery from these complications.

Studies have shown that there are significant differences in the antioxidant capacity of different types of infant feeding. One study has shown that the total antioxidant capacity in the breast milk of mothers who deliver prematurely is higher than the breast milk of mothers who deliver at term [14], while another study has found them to be equal [15]. However, both have superior antioxidant capacity when compared to formula [15,16]. Breast-fed and formula-fed infants show significant differences in plasma antioxidant nutrient concentrations [17]. The mother’s diet also affects the antioxidant capacity of human milk. An increased consumption of dairy products, fruits and vegetables, cereals and nuts has been shown to increase the total antioxidant capacity of the breastmilk [16].

Major nutritional antioxidants include α- and β-carotenes, lutein + zeaxanthin, lycopene, and α-tocopherol. Humans cannot synthesize these compounds and thus they must be provided exogenously through dietary intake. Carotene levels in colostrum have been shown to be five times higher than in mature breast milk [17]. Similarly, breast-fed premature infants have been shown to have higher serum carotenoids than formula-fed premature infants [18]. In one study, carotenoid supplementation was associated with a blunted increase in C-reactive protein (CRP) concentrations from one to 40 weeks post-menstrual age, whereas CRP levels rose in controls [19]. The association of a lower CRP with higher carotenoid consumption likely reflects carotenoid antioxidant and immunomodulatory properties. In populations of children with acute infections, a significant inverse correlation was shown between serum CRP and carotene concentrations [20]. Plasma β-carotene concentrations have indeed been found to be lower in infants with bronchopulmonary dysplasia [21], which may result in a reduction of their antioxidant protection. Our study does report that β-carotene concentrations in donor milk were less than one-third of those in fresh breast milk, and our *p*-value of 0.13, which approaches statistical significance, may be more likely due to the limited power of our study. This may indicate that further investigation into carotenoid anti-inflammatory effects in sick, preterm infants is warranted.

It is thought that lutein + zeaxanthin influence the maturation of cells in the macular region of the retina [22] and protect against stress and oxidation in the retinal pigment epithelium [23]. Vishwanathan et al. determined that the mean concentration of lutein was significantly greater than the other carotenoids in brain tissue samples of infants who died within the first 18 months of life [22]. Preterm infants also had significantly lower concentrations of lutein + zeaxanthin compared to term infants in most of the brain regions [22]. These findings, in addition to previous research, help support the role lutein + zeaxanthin plays in visual and cognitive development. Breast-fed infants have been shown to have higher serum lutein levels than formula-fed infants, possibly due to increased bioavailability of the compound in breast milk, and a dose-dependent relationship exists between lutein in the diet and lutein in the serum [24]. It was calculated that four times more lutein is needed in infant formula than in human milk to achieve similar serum lutein concentrations among breast-fed and formula-fed infants [24]. In a recent pilot randomized controlled trial in healthy newborns, lutein administration proved effective in increasing the levels of biological antioxidant potential by decreasing the total hydroperoxides as markers of oxidative stress [25]. In another study by Mazoni et al., the effect of lutein + zeaxanthin on prevention of BPD appears relevant, although not statistically significant (*p* = 0.07) [26]. Lutein supplementation also has been shown to result in greater rod photoreceptor sensitivity responses when compared to controls [19]. A pilot study showed a potential antioxidant effect of lutein in the neonatal period [25]; however, another study showed that lutein supplementation did not enhance the biological antioxidant capacity [27], although this second study did not achieve a statistically significant difference in the serum lutein concentrations between the placebo and intervention group. A positive association was seen between plasma lutein levels and total antioxidant status (*r* = 0.13, *p* = 0.02) [27]. Our study finds that concentrations of lutein + zeaxanthin were approximately half of the concentrations found in maternal breast milk. Both donor milk and maternal breast milk had lower lutein + zeaxanthin concentrations when compared to the infant formulas tested; however, given the study that demonstrated possible improved bioavailability from breast milk [24], it cannot be assumed that the formula-fed infants would have higher serum concentrations. Additionally, it is important to note that not all formula manufacturers provide supplemental lutein in preterm formula, and therefore infant intake may vary widely based on formula selection.

Vitamin E is an antioxidant that protects cell membranes against free radicals [28]. Although vitamin E deficiency is thought to be rare in healthy adults, it is much more common in premature infants [29]. Vitamin E occurs naturally in several different isoforms, including α- and γ-tocopherol. These isoforms differ by one methyl group and are not interconvertible in human metabolism [30]. As a result, increased intakes of α- or γ-tocopherol will cause a rise in serum concentrations of that specific tocopherol [31,32]. Importantly, serum and tissue levels of vitamin E isoforms correlate [33], meaning the dietary intake of tocopherols has the potential to influence biological mechanisms.

One change in infant nutrition that has occurred in the last several decades is the increase in the γ-tocopherol isoform in the diet of infants. This is primarily due to in the use of soy oils, which are extremely high in γ-tocopherol, as the primary lipid component in infant formulas [34]. While human breast milk has been shown to contain some γ-tocopherol, previous studies have shown the content of γ-tocopherol in infant formula to be up to seven times higher than that in human milk [35]. Our findings concur with this report, demonstrating γ-tocopherol levels in our formula samples to be 3.5–5.6 times higher than in maternal breast milk samples. This level of γ-tocopherol in infant formula does not appear to provide similar protection from lipid peroxidation as human milk [36]. Serum levels of γ-tocopherol in infants have been shown to increase during the first week of life [37], presumably from dietary sources [35]. Recently, our understanding of these tocopherols isoforms has expanded as new evidence indicates that vitamin E isoforms have different roles in influencing inflammation. In contrast to the anti-inflammatory properties of the α-tocopherol isoform, the γ-tocopherol isoform has been shown to increase cytokine production (i.e., IL-2) and demonstrate pro-inflammatory properties [38,39,40,41,42]. Importantly, serum γ-tocopherol isoforms at as little as 10% of the concentration of α-tocopherol have been shown to ablate the anti-inflammatory benefit of alpha-tocopherol [41]. With regard to α-tocopherol, long-term supplementation (six months minimum and up to 24 months) has been shown to positively impact mental development, particularly intelligence quocient (IQ), in school-age children who were extremely low-birth-weight infants (ELBW) [43], raising the possibility that α-tocopherol might be a functional molecule in a developing brain. Although some NICU infants receive donor milk for only a limited amount of time, other institutions use donor milk as a primary source of nutrition throughout NICU hospitalization; our finding that α-tocopherol levels in donor milk samples were significantly decreased when compared to breast milk may make consideration of vitamin E status important in these infants.

Donor milk still has unique advantages compared to formula and continues to represent an important alternative if maternal milk is not available, specifically with regard to necrotizing enterocolitis [44]. In a Cochrane systematic review and meta-analysis, Quigley et al. demonstrated both benefits and risks associated with the use of donor milk. Importantly, there was a higher incidence of NEC among infants with birth weights <2500 g and in those fed formula versus those fed donor milk (relative risk of 2.5 (95% CI, 1.2, 5.1) [44]. Because NEC is the most common gastrointestinal emergency among very-low-birth-weight (VLBW) infants, its prevention is a powerful argument in favor of donor milk as an alternative supplement to formula when the mother’s own milk is not available. The Quigley et al. review and meta-analysis, however, concluded that infants fed donor milk experienced slower weight (*p* < 0.0001), length (*p* < 0.0003) and head circumference (*p* < 0.0001) gains than those fed formula [44]. These risks associated with donor milk are of significant concern because VLBW infants are born with impoverished nutrient reserves, and are subject to metabolic stresses that further elevate nutritional requirements [45]. Nutrient deficits and sub-optimal growth have significant long-term neurodevelopmental consequences [46,47]. Quigley et al. point out that all but one of the randomized controlled trials examined in their meta-analysis were >25 years old, when smaller VLBW infants did not survive, and these studies also may not be reflective of current practice [44]. In a more recent study conducted in 2012–2014, The Early Nutrition Study Randomized Clinical Trial found no difference in infections, necrotizing enterocolitis, or mortality during the first 60 days of life in 373 infants fed pasteurized donor milk or preterm formula for supplemental feedings [48]. Another study 2014 of 201 ELBW infants found no difference in NEC or infection rates between infants receiving human milk (including donor milk) and infants receiving formula; however, the duration of mechanical ventilation was significantly higher among formula-fed infants (24 vs. 60 h, *p* = 0.016) in the group exposed to formula [48].

Our analysis has several limitations. First, the antioxidant capacity of breast milk includes many other compounds than the ones highlighted in this study, including uric acid, enzymes, and lipids. However, the antioxidants targeted in this study are modifiable by maternal diet, which may allow for interventions targeted at increasing the antioxidant potential of human milk. Additionally, we did not have the serum levels of donor milk–fed infants to compare to infants receiving maternal breast milk to compare the impact of decreased intake. Additionally, our statistical power was limited by low numbers of analyzed breast milk, a precious commodity to a premature neonate. The median gestational age of 37 weeks in our cohort includes preterm breast milk samples, and the donor milk samples are from a commercial pooled supply. Future studies evaluating the serum nutritional antioxidant status of infants receiving the mother’s own milk, donor milk, and infant formulas will expand our knowledge in this area.

## 5. Conclusions

As donor milk is becoming a common nutritional intervention for the high risk preterm infant, the nutritional antioxidant status of donor milk–fed premature infants and outcomes related to oxidative stress may merit further investigation.

## Figures and Tables

**Table 1:** Nutrition antioxidant content of infant feedings.

Nutritional Antioxidant (µg/L)	Premature Formula	Transitional Formula	Term Standard Formula	Breast Milk Mean (SD) *N* = 12	Donor Milk
α-carotene	0.51	1.40	0.5	7.7 (14.5)	3.6
β-carotene	71.1	63.9	25.0	49.1 (75.5)	13.7
β-cryptoxanthin	0.9	0.9	0.48	21.7 (40.0)	3.8
Lycopene	1.5	5.8	79.8	66.1 (55.9)	11.9
Lutein + zeaxanthin	65.5	56.9	58.4	40.1 (42.5)	21.4
Retinol	3086.2	911.8	571.2	401.6 (516.3)	185.8
α-tocopherol	20,109.1	13,360.2	8520.0	5880.8 (4971.7)	1381.9
γ-tocopherol	6787.1	6561.6	4204.0	1207.1 (668.4)	622.8
